# Fertility Enhancing Activities of Bioactive Components of Cochlospermum planchonii Rhizome on Cisplatin Induced Reproductive Dysfunctions in Sprague–Dawley Rats 

**Published:** 2018-09

**Authors:** Sunday A. Adelakun, Busuyi K. Akinola, Grace T. Akingbade

**Affiliations:** 1 Department of Human Anatomy, School of Health and Health Technology, Federal University of Technology, Akure, Ondo State, Nigeria; 2 Department of Anatomy, Faculty of Basic Medical Sciences, Ladoke Akintola University of Technology, Ogbomoso, Oyo state, Nigeria

**Keywords:** Cisplatin, Cochlospermum Planchonii, Oxidative Stress, Hormone, Rats, Fertility

## Abstract

**Objective:** Cisplatin has been established to cause reproductive dysfunction; Cochlospermum planchonii is globally used in folklore medicine and has numerous therapeutic benefits. This study focused on fertility enhancing activities of *Cochlospermum planchonii* (Cp) on cisplatin-induced reproductive dysfunctions.

**Materials and methods:** Total of 30 male and 30 female adult Sprague-Dawley rats were used for this study. The male rats randomly assigned into Group A (control) was given normal saline 2 ml/kg, Group B, C, D and E rats received(single dose of 8 mg/kg Cisplatin (i.p.) on the first day), (500 mg/kg body weight (bwt) of *Cp*once once daily for 14 days), (single dose of 8 mg/kg Cisplatin (i.p.) on 1^st^ day followed by 500 mg/kg bwt of *Cp *once daily for 14 days) and (single dose of 8 mg/kg Cisplatin on first day followed by 50 mg/kg vitamin C for 14 days). Parameters tested include reproductive hormones, testicular histology, testicular antioxidants, semen parameters and fertility test.

**Results:** Histological profile of the testes revealed derangement of the testis cytoarchitecture; Seminiferous epithelium, body, testes, accessory sex organs weight and sperm parameters, were significantly reduced (p <0.05). Hormonal assay showed significant changes in testosterone (p< 0.05) while luteinizing hormone and follicle stimulating hormone remained unchanged following cisplatin administration and a marked improvement was observed after *Cochlospermum planchonii* administration. Similarly, *Cochlospermum planchonii *improved the reduction of antioxidant parameters (SOD, CAT, GPx and GSH) and the increased MDA caused by cisplatin ingestion.

**Conclusion:** *Cochlospermum planchonii *may thus offer protection against free radical mediated oxidative stress of rats with cisplatin induced reproductive dysfunction.

## Introduction

Infertility is one of the most controversial health issues in medical sciences (1). Male factors, including hormonal problems, decreased sperm quality and quantity are the cause of 30% of cases reported of infertility (2). Several diseases, such as coronary heart diseases, diabetes mellitus and chronic liver diseases may interfere with the spermatogenesis process, and therefore sperm quality and quantity may be altered by these diseases (3). Cisplatin is an established chemotherapeutic substance commonly used in the treatment of cancer (4) or diverse types of solid tumors (5). Cisplatin has been successfully used to treat lung cancer, squamous cell cancer of the head and neck, and especially testicular cancer (6, 7). However, Cisplatin has some side effects; High dosage of cisplatin can lead to nephrotoxicity and hepatotoxicity (8). Cisplatin can induce placental hypoplasia and pass through the placental barrier in pregnant rats (9). Moreover, cisplatin can cause haemodynamic changes and lesions to myocardial cell mitochondria through oxidative stress, inflammation, and apoptosis in rats (10). Despite the improvement in quality of life of cancer patients; the use of cisplatin was restricted clinically by its major side effects including testicular toxicity (11, 12). In adult men, fertility can be preserved by spermatozoa cryopreservation and intracytoplasmic sperm injection; however, these methods are not feasible options for pre-pubertal patients (13). Besides, in adults, the freezing and thawing of semen can reduce the sperm quality (14). Interestingly, growing evidences revealed that combination therapy can be beneficial to overcome this special reproductive toxicity (15, 16). Male reproductive toxicity rises from targeting rapidly growing testicular cell types such as Leydig, Sertoli, and germ cells (17). Cisplatin adversely impairs non target tissues via increased free radical generation and depletion of antioxidants; which results in inhibition of protein synthesis and DNA damage (11, 18). Oxidative stress is an important process that is involved in multiple conditions like infertility and inflammation (19). Therefore, these diseases are controlled in people receiving antioxidant supplements (20). Antioxidants are regarded as significant agents, which contribute to the overall health of the organism. Polyphenols, as dietary antioxidants, are associated with redox activities and have beneficial effects on health (20). 

*Cochlospermum planchonii (*Cochlospermaceae) is a bushy plant that is widespread in the savannah and shrub land of West Africa with bright yellow flowers of about 2.5- 3 m in height. The plant is a common traditional medicine in Burkina Faso, Cameroon, Gambia, Ghana, Guinea, Ivory Coast, Nigeria and Senegal (21). In Nigeria, it is called “N′ Dribala” (Fulani), “Rawaya” or “Kyamba” (Hausa), “Abanzi” (Igbo) and “Gbehutu or Feru” (Yoruba). The root decoction is most frequently used for treatment of infertility, premenstrual pain, gonorrhea (21) and diabetes mellitus (22) and the leaves are used to treat jaundice, malaria (23) and diarrhea (24). The leaf, stem bark and root bark of *Cochlospermum planchonii *have been reported to have strong antifungal effect against *Colletotrichum capsici* (25). In Northern Sierra Leone, the root decoction has been reported to be effective in the treatment of gonorrhea, whereas the stem decoction is used in the treatment of menstrual disorders (26). The trypanocidal activity of chloroform extract of the stem bark of *Cochlospermum planchonii *had been reported (27). Bioactive compounds of the methanolic root extract of *Cochlospermum planchonii *are reported to cause central nervous system (CNS) depression, possess analgesic, anti-inflammatory and anti-diabetic activities (28). 

Present study focused on Fertility enhancing Activities of bioactive component of *Cochlospermum planchonii Rhizome*on Cisplatin-induced reproductive dysfunctions in Sprague Dawley rats.

## Materials and methods

***Chemicals and Drugs:*** Cisplatin (Medrel pharmaceuticals, India) and vitamin C (Emzor Pharmaceuticals, Nigeria) were obtained from the Department of Pharmacy of the State Specialist Hospital, Akure, Ondo State Nigeria, immunoassay kit obtained from Randox United Kingdom and all other chemicals were of the highest purity and analytical grade.

***Plant Material:*** Plant materials were collected from the Research Farm, Faculty of Agricultural Sciences, LadokeAkintola University of Technology (LAUTECH) Ogbomoso, Oyo State, Nigeria. Samples of *Cochlospermum planchonii *roots were identified and authenticated by Prof. A.T.J. Ogunkunle of the Department of Pure and Applied Biology and sample of the plant voucher deposited for reference purpose.

***Extraction of plant material:*** The roots were thoroughly washed in sterile water and air dried under shade for two weeks to a constant weight in the laboratory. The air-dried roots were weighed using CAMRY (EK5055, Indian) electronic weighing balance and were milled with automatic electrical Blender (model FS-323, China) to powdered form. Five hundred grams of the milled plant sample was later soaked in 1000 ml of phosphate -buffered saline (PBS) for 48 hours (29) at room temperature, and was later filtered through cheese cloth and then through What man #1 filter paper (30), the filtrate was concentrated using a rotary evaporator (Rotavapor® R-220) at 42- 47°C.

***Phytochemical screening:*** Qualitative phytochemical analysis of the extract of *Cochlospermum planchonii *roots was done in accordance with Soni and Sosa (31).

***Animals:*** Male and female Sprague-Dawley rats were procured from the Experimental Animal House Department of Anatomy, Ladoke Akintola University of Technology, Ogbomoso, Nigeria. The animals were kept in cages and allowed to acclimatize for a period of two weeks before the commencement of the experiment. The rats were maintained under standard natural photoperiodic condition of twelve hours of darkness and twelve hours of lightness (D:L; 12:12h dark/light cycle) at room temperature (25-32^0^C) and humidity of 50-55% (32). Their cages were cleaned every day. The rats were fed with standard rat chow at a recommended dose of 100 g/kg daily as advised by the International Centre of Diarrheal Disease Research; Bangladesh (ICDDR, B).Drinking water was supplied *ad libitum*. The weight of the rats were documented at procurement, during the period of acclimatization, at commencement of administrations and once a week throughout the period of the experiment, using an electronic analytical and precision balance CAMRY (EK5055, Indian). 

***Experiment design and animal grouping:*** A total of 30 male and 30 female healthy adult (12-14 weeks old) Sprague-Dawley rats weight 200 ± 20g were used for this study. The male rats were randomly divided into four groups (A, B, C, D and E) of six (n = 6) rats each. Group A which served as control were given normal saline 2 ml/kg each daily for 14 days. Group B, C, D and E rats were administered with (single dose of 8 mg/kg Cisplatin (i.p.) on the first day), (500 mg/kg body weight of *Cochlospermum planchonii *once daily for 14 days), (single dose of 8 mg/kg Cisplatin (i.p.) on the 1^st^ day followed by 500 mg/kg body weight of *Cochlospermum planchonii *once daily for 14 days) and (single dose of 8 mg/kg Cisplatin on the first day followed by 50 mg/kg vitamin C as stardand drug for 14 days). Female rats were used to copulate with the control and treated male rats to test for fertility after the administration. At the end of the treatment, rats were weighed, blood samples were collected through orbital venous sinus of live animals with microhematocrit tubes within the hours of 7:00 am and 8:00 am and immediately centrifuged at 3000 xg for 10 minutes for serum separation to estimate reproductive hormone. Thereafter, animals were euthanized and the reproductive organs were immediately removed, cleaned from the adhering tissue, and weighed. The epididymal content for each rat was instantly collected for semen analysis. Furthermore, the left testis was fixed in Bouin‘s fluid for histological procedure. Whereas, the right testis was decapsulated, homogenized in 0.05 M potassium phosphate buffer (pH 7.4), and processed for the estimation of oxidative satures. All samples were stored at -80°C until analysis.

***Measurement of sperm parameters:*** The rats were anaesthetized with diethyl ether. A scrotal incision was made to exteriorize the testis and epididymides. The caudal epididymis was carefully removed, blotted free of blood and then placed in a prewarmed Petri dish containing 2.0 ml of medium Ham's F-10 medium (Sigma, USA) containing 0.5% bovine serum albumin. After 5 minutes of incubation at 37°C (with 5% CO2). Several incisions were made on it to allow sperm swim out. Semen analysis was carried out immediately using the new improved Neubauer’s haemocytometer counting chamber for determination of the concentration of spermatozoa. Sperm motility was also assessed immediately by counting both motile and immotile spermatozoa per unit area at the magnification of 40x according to the World Health Organization (33) method. Sperm viability was assessed using eosin-nigrosin test. The percentages of unstained (live) and stained (dead) spermatozoa were calculated by counting 200 spermatozoa per sample. Morphological appearance of normal and abnormal spermatozoa was determined by examining stained smears under the oil immersion (100x) and their percentages were calculated.

***Hormone determination*** *:* The serum levels of Testosterone (TT), Follicule stimulating hormone (FSH) and Leutenizing hormone (LH) were measured using commercially available enzyme-linked immunoassay kit (Diagnostic automation Inc, CA)obtained from Randox Laboratories Ltd., Admore Diamond Road, Crumlin, Co., Antrim, United Kingdom, according to the manufacturer’s instructions.

***Biochemical estimations:*** Testicular homogenate was divided into 3 aliquots, where the first one was used for estimation of malondialdehyde (MDA) (34). The second aliquot was deproteinized with 5% sulfosalicylic acid, centrifuged at 1000 xg for 15 minutes and the obtained supernatant was used for the estimation of reduced glutathione (GSH) (35). The third one was centrifuged at 4000 rpm for 15 minutes at 4°C and the resultant supernatant was used for the assay of superoxide dismutase (SOD), catalase (CAT) and glutathione peroxidase (GPx),

***Testicular histology preparation:*** The histology of the testes was done by modification of method reported by Kayode *et al.* (36). The organs were harvested and fixed in Bouin‘s fluid for 24 h after which it was transferred to 70% alcohol for dehydration. The tissues were passed through 90% and absolute alcohol and xylene for different durations before they were transferred into two changes of molten paraffin wax for 1 hour each inan oven at 65◦C for infiltration. They were subsequently embedded and serial sections cut using rotary microtome at 5 microns. The tissues were picked up with albumenized slides and allowed to dry on hot plate for 2 min. The slides were dewaxed with xyleneand passed through absolute alcohol (2 changes); 70% alcohol, 50% alcohol and then to water for 5 min. The slides were then stained with haematoxylin and eosin. The slides were mounted in DPX. Photo micrographs were taken at a magnification of x100

***Morphometry:*** Morphometric analysis was done using Leica DM750 microscope (Germany) in the Department of Anatomy, college of Medicine, University of Lagos, Nigeria. The primary aim was to estimate the volumes of seminiferous tubule epithelium (seminiferous epithelium) and interstitium in the testis. This was done in accordance with Howard and Reed (37) and Baines *et al. *(38). Four sections per testis, and six microscopical fields per section, were randomly chosen for analysis. Fields were sampled as images captured on a Leica DM750 bright field microscope (Germany) via LAZ software. Volume densities of testicular ingredients were determined by randomly superimposing atransparent grid comprising 35 test points arranged in a quadratic array. Test points falling on a given testis and its ingredients were summed over all fields from all sections. The total number of point sitting on a given ingredient lumen (EL), epithelium (EE), interstitium (EI), divided by the total number of points hitting on the testis sections (ET) multiplied by 100, provided an unbiased estimate of its % volume density/volume fraction.

***Fertility Test:*** The fertility test was done by modification of method reported by Ligha *et al. *(39). Each male rat was isolated and paired with a pro-oestrous female rat in the first hours of oestrous cycle determined by vaginal smear examination and placed in a single cage with each male rat’s 1:1. On the following day, the female rats were checked after mating to detect spermatozoa in their vagina by microscopic examination of the vaginal fluid. Females in which spermatozoa plug were detected the following morning after mating represented day one of gestation. The fetuses were removed by ventral laparotomy on the 21^st^ day of gestation. The foetus was counted.

***Ethical considerations*** *:* All experimental procedures followed the recommendations provided in the “Guide for the Care and Use of Laboratory Animals” prepared by the National Academy of Sciences and Published by the National Institute of Health (40).

***Data presentation and statistical analysis:*** Data were expressed as Mean± SEM. Statistical differences between the groups were evaluated by one way ANOVA, followed by Newman-Keuls Multiple Comparison Test. Differences yielding p < 0.05 were considered statistically significant. Statistical analyses of data were performed using Graph Pad Prism 5 Windows (Graph Pad Software, San Diego, California, USA).

## Results

***Phyto chemical screening:*** Qualitative analysis of *Cochlospermum planchonii* root revealed bioactive constituents present in the extract such as flavonoids, Saponins, Steroids, Terpenoids, Tannins, and Glycosides (Table 1).

**Table 1:** Qualitative phytochemical analysis of the ethanolic extract of root extract of Cochlospermum planchonii

**S/N**	**Phytochemicals**	**Results**
1	Flavonoids	+
2	Saponins	+
3	Steroids	+
4	Terpenoids	+
5	Tannins	+
6	Cardiac glycosides	+
7	Phlobatannins	-
8	Alkaloids	-
9	Quinones	-
10	Coumerins	-

+ Present and – not present

***Change in body and organs weight:*** There was no significant change in body and organs weight between the control rats and those which received *Cochlospermum planchonii* alone and cisplatin plus vitamin C at the selected doses. 

**Table 2:** Activities of root extract of Cochlospermum planchonii on body and reproductive organ weights of cisplatin-induced reproductive dysfunctions in Sprague–Dawley rats

**Parameters **	**Groups**				
	**A(control)**	**B(Cisplastin)**	**C(C.** ***planchonii*** **)**	**D(Cisplastin + ** **C.** ***planchonii*** ** )**	**E(Cisplastin + ** **Vitamin C)**
Initial body weight (g)	211.4 ± 3.86	212.8 ± 2.92	209.3 ± 2.94	211.6 ± 1.65	211.1 ± 3.03
Final body weight (g)	271.5 ± 5.38	216.5 ± 0.91*	270.7 ± 6.78α	268.6 ± 4.63α	259.3 ± 1.88α
Absolute Testes weight (g)	1.98 ± 0.05	0.50 ± 0.04*	1.89 ± 0.06	1.92 ± 0.05α	1.09 ± 0.14*^,^α
Absolute Epididymis weight (g)	0.39 ± 0.023	0.12 ± 0.026*	0.36 ± 0.017	0.37 ± 0.022α	0.25 ± 0.016*^,^α
Absolute ventral prostate weight (g)	0.42 ± 0.028	0.18 ± 0.011*	0.48 ± 0.019	0.45 ± 0.020α	0.39 ± 0.010*^,^α
Absolute seminal vesicle weight (g)	0.49 ± 0.024	0.21 ± 0.017*	0.55 ± 0.028*	0.54 ± 0.026α	0.43 ± 0.020α
Absolute vas deferens weight (g)	0.15 ± 0.008	0.10 ± 0.006*	0.15 ± 0.009	0.16 ± 0.010α	0.14 ± 0.006α

Values are expressed as Mean ± S.E.M; n = 6 in each group;

* represent significant different from control;

α represent significant different from group B at p < 0.05; One-Way ANOVA.

Administration of *Cochlospermum planchonii* alone significant (p < 0.05) increased absolute weight of seminal vesicle (0.55 ± 0.028) in comparison with control (0.49 ± 0.024), also there was significant (p < 0.05) increased in body and organs weight in all the treatment group when compared with the group B that received single dose of 8 mg/kg Cisplatin (i.p.) on the first day. However a significant decreased in body as well as absolute reproductive organs weight was observed as a result of cisplatin administration compared to control group. Also the weight of testis, cauda epididymis, prostate, seminal vesicle and vas deferens were significantly decreased after cisplatin exposure when compared to that of the control group while administration *Cochlospermum planchonii *and vitamin C restored the reproductive organs weight (Table 2).

***Sperm parameters: ***There was significant (p < 0.05) decreased in mean value of sperm motility, count and viability of cisplatin treated rats (35.20 ± 4.10, 36.73 ± 4.06, 37.02 ± 4.93) and an increased in sperm abnormalities (61.21 ± 1.06) compared to the corresponding values of the control group (80.14 ± 5.39, 84.61 ± 4.07, 81.88 ± 4.30) and (12.72 ± 1.01). Treatment with *Cochlospermum planchonii *and vitamin C alleviated the changes in motility, count, viability and sperm abnormalities meanwhile, *Cochlospermum planchonii *administration significantly increased sperm count, motility and reduced sperm abnormalities in comparison with cisplatin group. Also the extract caused significant (p < 0.05) increases in sperm motility, sperm count and viability of extract treated rats. Furthermore, the percentages of abnormal sperm cells (morphology) in *Cochlospermum planchonii *and vitamin C treatment groups were not significantly different from the control but significant (p < 0.05) dissimilar from the cisplatin treated group (Table 3).

***Antioxidant Levels (CAT, SOD, GSH, GPx) and MDA Levels:*** As showed in table 4, the mean value of tMDA levels in the cisplatin group significantly (p < 0.05) increased (18.40 ± 1.36) compared with the control group (6.03 ± 0.78) but decrease in Cisplatin + *Cochlospermum planchonii*, Cisplatin + vitamin C and *Cochlospermum planchonii* group (8.43 ± 0.77, 11.92 ± 0.80) and (5.51 ± 1.07) compared to the cisplatin group (18.40 ± 1.36).

**Table 3:** Activities of rootextract of Cochlospermum planchonii on sperm parameters of cisplatin-induced reproductive dysfunctions in Sprague–Dawley rats

**Groups **	**Parameters**				
	**Sperm motility (%)**	**Sperm court (x 10** ^6 ^ **/ml)**	**Viability (%)**	**Morphology (%)**	
				**Normal**	**Abnormal**
A (control)	80.14 ± 5.39	84.61 ± 4.07	81.88 ± 4.30	87.28 ± 1.03	12.72 ± 1.01
B (Cisplastin)	35.20 ± 4.10*	36.73 ± 4.06*	37.02 ± 4.93*	38.79 ± 3.21*	61.21 ± 1.06*
C (C.*planchonii*)	76.91 ± 4.05 α	83.84 ± 4.30α	79.39 ± 4.44α	85.35 ± 1.73α	14.65 ± 2.04α
D (Cisplastin + C.*planchonii*)	75.82±3.83α	75.36 ± 5.08α	75.92 ± 5.45α	83.94 ± 1.75α	16.06 ± 1.62α
E (Cisplastin + Vitamin C)	54.24±3.45*^,^α	57.10 ± 3.33*	59.18 ± 5.81*	75.24 ± 2.35α	24.76 ± 1.82*^,^α

Values are expressed as Mean ± S.E.M; n = 6 in each group;

* represent significant different from control;

α represent significant different from group B at p < 0.05; One-Way ANOVA.

**Table 4:** Activities of root extract of Cochlospermum planchonii on testicular antioxidant levels and lipid peroxidation levels in rat

**Parameters**	**Groups**				
	**A(control)**	**B(Cisplastin)**	**C(C.** ***planchonii*** **)**	**D(Cisplastin + ** **C.** ***planchonii*** ** )**	**E(Cisplastin + Vitamin ** **C)**
tMDA (nmol/min)	6.03 ± 0.78	18.40 ± 1.36*	5.51 ± 1.07α	8.43 ± 0.77α	11.92 ± 0.80*^,^α
tSOD (U/g protein)	12.11 ± 2.10	5.43 ± 0.93*	11.55 ± 1.41 α	10.83 ± 1.20α	8.37 ± 1.53
tCAT (lmol/mg protein)	17.50 ± 2.08	7.28 ± 1.33*	14.38 ± 1.18 α	13.46 ± 1.77α	10.34 ± 1.42*
tGSH (lmol/min)	10.37 ± 1.13	4.51 ± 0.76*	10.43 ± 1.52 α	9.68 ± 0.89α	6.21 ± 0.76
tGPx (lmol/min)	6.17 ± 0.97	2.43 ± 0.63*	7.74 ± 0.81 α	5.19 ± 0.62α	4.00 ± 0.50

Values are expressed as Mean ± S.E.M; n = 6 in each group;

* represent significant different from control;

α represent significant different from group B at p < 0.05; One-Way ANOVA.

The anti-oxidant levels (tCAT, tSOD, tGSH and tGPx) (7.28 ± 1.33, 5.43 ± 0.93, 4.51 ± 0.76 and 2.43 ± 0.63) decreased significantly in cisplatin group (p < 0.05) compared to the control group (17.50 ± 2.08, 12.11 ± 2.10, 10.37 ± 1.13 and 6.17 ± 0.97) but the tCAT, tSOD, tGSH and tGPx levels in Cisplatin + *Cochlospermum planchonii *Cisplatin + vitamin C and *Cochlospermum planchonii *group decreases compared to the control group.

***Hormonal assay and Morphometric (stereological) analysis:*** Compared with the control, animals in the Cisplatingroup had significantly reduced TT, LH and FSH (p < 0.05) levels. In the Cisplatin + *Cochlospermum planchonii*, Cisplatin + vitamin C and *Cochlospermum planchonii* group, TT levels as well as FSH and LH levels were not significantly different from the values of control (Table 5). At the end of this study the volume density of the germinal epithelium of group A had a significant difference with group B. Also, there was a significant increase in groups C, D and E when compared to groups A and B. The lumen density significantly decreased in group B compared to group A. The interstitium had a significant increase in group B compared to group A. There was also a significant decrease in the interstitium of groups C, D and E compared to group B (Table 5).

***Fertility Test:*** In group B treated with single dose of 8 mg/kg Cisplatin (i.p.) on the first day observed to have adverse effect on the fertility potentials of treated male rats since the female rats proven infertility copulated with the treated male rats did not pregnant. Whereas in group D co-treated with Cisplatin and *Cochlospermum planchonii *was found not to have negative effects on fertility potentials of treated male rats since all the female rats mated with the treated male rats got pregnant and produced at least six fetuses. There was significant decreased in number of fetuses produced in group E treated with Cisplatin+ vitamin C (p < 0.05) when compared with the control group. In experimental group treated 500 mg/kg body weight *Cochlospermum planchonii* extract produced more number of fetuses compared with group B and group E (Table 6).

***Testicular Histology*** *:* Sections of the seminiferous tubules of the control rats were moderately circular or oval in outline with normal stratified seminiferous epithelium showing cells of the spermatogenic series and spermatozoa within the lumen also the seminiferous tubules illustrating all the stages of spermatogenesis from spermatogonia to mature sperm cells.

**Table 5:** Activities of root extract of Cochlospermum planchonii on testosterone level, FSH and LH and morphometric parameters of testis

**Parameters **	**Groups**				
	**A(control)**	**B(Cisplastin)**	**C(C.** ***planchonii*** **)**	**D(Cisplastin + ** **C.** ***planchonii*** ** )**	**E(Cisplastin + Vitamin ** **C)**
Testosterone (ng/ml)	4.95 ± 0.37	2.10 ± 0.36*	4.71 ± 0.29α	4.85 ± 0.40α	3.96 ± 0.42α
FSH (mlu/ml)	3.60 ± 0.32	1.58 ± 0.14*	2.95 ± 0.30α	2.92 ± 0.18α	2.68 ± 0.17α
LH (mlu/ml)	32.21 ± 3.42	19.08 ± 1.93*	39.04 ± 3.55α	19.47 ± 1.94	19.18 ± 1.92*
Germinal epithelium (%)	64.60 ± 1.30	60.50 ± 1.18*	70.29 ± 0.88* α	69.38 ± 0.78* α	70.84 ± 1.78* α
Lumen (%)	14.67 ± 0.42	10.17 ± 0.28*	11.98 ± 0.52α	11.52 ± 0.32*	10.99 ± 0.50*
Interstitium (%)	20.14 ± 1.02	25.49 ± 1.84*	17.70 ± 0.74α	15.87 ± 0.76α	18.20 ± 0.72α

Values are expressed as Mean ± S.E.M; n = 6 in each group;

* represent significant different from control;

α represent significant different from group B at p< 0.05; One-Way ANOVA.

**Table 6:** Fertility test in female rats

**Parameters **	**Groups**				
	**A (control)**	**B (Cisplastin)**	**C ** **(C.** ***planchonii*** **)**	**D (Cisplastin + ** **C.** ***planchonii*** ** )**	**E (Cisplastin + Vitamin C)**
No. of pregnancy	6.00	0.00	6.00	6.00	4.00
No. of fetus	9.00 ± 0.97	0.00 ± 0.00*	10.17 ± 0.95α	8.33 ± 0.07α	2.83 ± 0.95*^,^α

Values are expressed as Mean ± S.E.M; n = 6 in each group;

* represent significant different from control;

α represent significant different from group B at p < 0.05; One-Way ANOVA.

**Figure 1:**
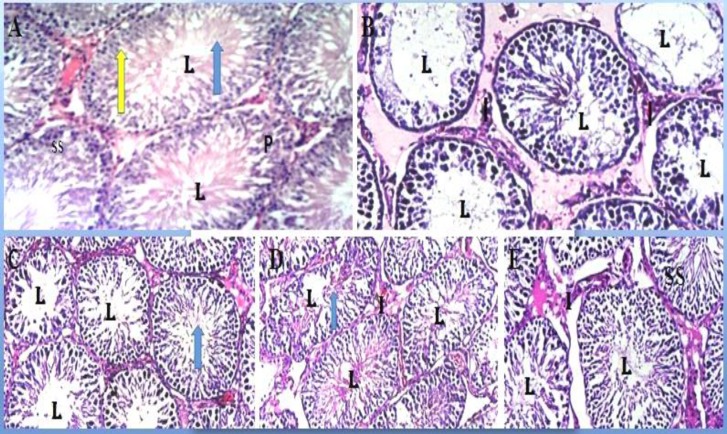
Photomicrographs of testis (H & E;×100) after administration. Group A (control) showing the seminiferous tubules containing cells of the spermatogenics eries (SS) and the lumen (L) containing spermatozoa; yellow arrow represents spermatogonium; P represents primary spermatocytes; blue arrow represents spermatids and spermatozoa. Group B (Cisplatin) showing hypocellularity and reduction in cellsof the spermatogenic series (SS). Group C–E showing normal cellularity in germinal epithelium, lumen filled with sperm cells andinterstitial cells of Leydig in the interstitium in groups C (500 mg/kg body weight of Cochlospermum planchonii), D (Cisplatin + 500 mg/kg body weight of Cochlospermum planchonii) and E (Cisplatin + 50 mg/kg vitamin C).

Testicular sections of the cisplatin group revealed severe degeneration in the seminiferous tubules, shrinkage in germ cell layers and disruption of spermatogenesis, hypocellularity in the interstitium, congestion in blood vessels and widening of tubular lumen, tubular atrophy and decreased spermatozoa in tubular lumen. Group C, D and E showed normal cellular composition with no obvious aberrations compared to group B that showed marked depletion of spermatogenic cells with a widened and a hypo-cellular interstitium (Figure 1).

## Discussion

The qualitative phytochemical screening of *Cochlospermum planchonii* root extract revealed bioactive constituents present in the extract such as flavonoid, terpenoids, saponin, tannins, steroid and cardiacglycoside which are antioxidant agent against factors causing inflammation, diabetes, cardiac failure, hypertention, bacterial infection, cancercells, diarrhea, scurvy and membrane lipid perioxidation as shown in Table 1 and reported by Vaidya *et al *(41). The above phytochemical findings provide information on the protective functions of *Cochlospermum planchonii* root extract against factors that might have implicated the testes to cause cell damage. Spermatogenic cells are quite susceptible to deteriorating effects of chemotherapeutic drugs and they can lead to infertility by causing irreparable damage in stem cell colonies (42). It has been reported that apoptosis induced in germ cells depend on the dose of administered cisplatin (43). Cisplatin treatment interfered with maturation process of cells in spermatogenic series, reduced the spermatogenesis, and influenced fertility by causing azoospermia (43). Azoospermia and long-lasting testicular atrophy are common adverse consequences of cancer treatment and chemotherapeutic agents may disrupt spermatogenesis by targeting various testicular cell types (Leydig cells, Sertoli cells, and germ cells) and by activating numerous molecular pathways involved in germ cell life-and-death decision making (44). In this study administration of cisplatin significantly decreased the body and absolute reproductive organs weight of the cisplatin treated animalsin comparison with the animals in control group, this is in line with the report of Dilek and Seren, (42) and Ahmed *et al. *(16). However administration of *Cochlospermum planchonii* root extract markedly increased body weights and reproductive organs of the animals. This is inconsonance with findings by Abu* et al.* (45). Androgens regulate the weight, size and secretory function of testes, epididymes and accessory organs (46). *Cochlospermum planchonii* root extract increased cellular activity in the testes. This support the findings of Shittu*et al*. (47) that says increased cellular activities are key factor to be considered in the evaluation of organ weights.

At the end of our study, cisplatin significantly reduced sperm count, sperm motility, viability and increased abnormal sperm morphology by subjecting the spermatozoa to increased oxidative stress-induced damage because their plasma membranes contain large quantities of polyunsaturated fatty acids (PUFAs) (48) and their cytoplasm contains low concentrations of scavenging enzymes (49). Increased formation of reactive oxygen species (ROS) has been correlated with a reduction of sperm motility (50). The link between ROS and reduced motility may be dueto a cascade of events that result in a rapid loss of intracellular ATP leading to axonemic damage and sperm immobilization (51). Co-treatment of cisplatin and *Cochlospermum planchonii *root extract prevented the ravaging effects of cisplatin on sperm count, sperm motility, viability and decreased abnormal sperm morphology. The finding from this study has shown that *Cochlospermum planchonii* is rich in antioxidant constituents such as flavonoid, terpenoids, saponin, tannins, steroid and cardiac glycoside. This is in concordance with reports of Abu* et al*. (45). Therefore, it is plausible deducing that the rich antioxidant *Cochlospermum planchonii *boosted the testicular non-enzymatic and enzymatic antioxidants to effectively scavenge the free radicals preventing lipid peroxidation. The consequence is hereby reflected in the increased sperm count, sperm motility, sperm viability and decreased abnormal sperm morphology. This finding is in concordance with the reports by Rodrigues *et al. *(52).

It was also observed that malondialdehyde (MDA) a by product of lipid peroxidation (49) was increased in the animals treated with single dose of 8 mg/kg Cisplatin (i.p.) on the first day. This depicts an increase in lipid peroxidation. This study further showed that an increased lipid peroxidation elicited an increased production of superoxide dismutase to neutralized the effects of the free radicals. The excess lipid peroxidation in the cisplatin treated groups as measured by the formation of thiobarbituric acid-reactive substances (TBARS) in the present study corroborates these findings. As a result, MDA level used to measure the degree of peroxidative damage sustained by the spermatozoa was significantly increased in these groups (Table 4) leading to impaired sperm function, decreased sperm motility (50, 53) and ultimately increased number of non-motile/dead spermatozoa as observed in the present study. Indeed, loss of motility has been highly correlated with the lipid peroxidation status of the spermatozoa (54).

In addition, the antioxidant enzymes and lipid peroxidation levels can be used to predict the severity of cisplatin induced testicular damage. Antioxidants enzymes such as SOD, CAT, GSH and GPx dependently act in the metabolic pathways that involve free radicals. Therefore, decrease in the level of SOD, CAT, GSH and GPx in the testis suggests the toxic effects of cisplatinon testicular functions but the administration of *Cochlospermum planchonii* can counter the activities of cisplatin on testicular cells thereby blocking the decreased antioxidant levels. Since it was proved that the significance of GSH in the detoxification of chemically reactive metabolite in drug induced toxicity after decreased in GSH) (55, 56, 57) then we can deduce that increased oxidation and decrease synthesis of GSH causes decrease in GSH levels. Therefore increase in antioxidant enzyme activities levels (SOD, CAT, GSH and GPx) after *Cochlospermum planchonii *extracts administration might contribute to the ameliorating effects of oxidative stress.

The observed decrease in testosterone, luteinizing hormone and follicle stimulating hormone in the groups treated with single dose of 8 mg/kg Cisplatin (i.p.) on the first day in this study is in consonance with the report by Kilarkaje *et al*. (58). Cisplatin is known to adversely affect the production of testosterone by disrupting the hypothalamic-pituitary-testicular axis (59) through oxidative stress and inducing cellular toxicity (60). The suppression of LH led to the observed decrease in serum testosterone concentration and in turn decreased sperm concentration in the Cisplatin treated groups, the decrease in testosterone level might be the cause of decrease sperm count, motility, viability and increase abnormal morphology because testosterone is the principal hormone responsible for development of the spermatogonia stem cells to mature sperm cells (61, 62). The effect of cisplatino n FSH was however cushioned by 500 mg/kg body weight of *Cochlospermum planchonii *extracts; this may have also contributed to the improved sperm concentration observed in the cisplatin groups treated with 500 mg/kg body weight of *Cochlospermum planchonii *extracts. The exogenous antioxidants present in *Cochlospermum planchonii *may have largely contributed to the improved FSH level and sperm concentration level in the cisplatingroup treated with 500 mg/kg body weight of *Cochlospermum planchonii *extracts.

Our study revealed histological abnormalities in testicular tissue of animals in cisplatin treated group such as sloughing and shortening of seminiferous epithelium leading to reduction in cells of the spermatogenic series. Observed degeneration and atrophy of seminiferous tubules leading to reduction in volume density of the germinal epithelium, this is concert with study of Donmez and Bozdogan (4) and Ahmend *et al. *(57) who reported histological abnormalities in the testicular tissue of cisplatin treated animals. For decades, it has been made clear that testosterone which is produced by the interstitial cells of Leydig is a necessary prerequisite for the maintenance of established spermatogenesis (63). The reduced cellularity of the interstitium in testis of animals treated with only cisplatin would consequently lead to a decrease in testosterone resulting in the poor spermatogenesis observed. *Cochlospermum planchonii* root extracts maintained histoachitecture of the testis, increased the proliferative activity of spermatogonia and maintained the volume density of the interstitium compared to the control animals. From our observation, when *Cochlospermum planchonii* root extracts was co-administered with cisplatin; it protected the testis from the pernicious effects of cisplatin. This protective nature of *Cochlospermum planchonii *is enhanced by some of its phyto chemical constituents. It is plausible that the antioxidants militated against the ravaging effects of cisplatin on the testis. Moreover, Aitken and Roman (64) reported that flavonoids promote spermatogenesis. Hence, the increase in the volume density of the germinal epithelium of animals administered only with *Cochlospermum planchonii *is a consequence of the positive effects of its multivitamins and phytochemicals. In conclusion, cisplatin damage the germinal epithelium and depleted Sertoli cell, caused widening and hypo cellularity of the interstitium. *Cochlospermum planchonii *however, did not only promote germinal epithelial growth but protected the cyto architecture of the testis from the damaging effects of cisplatin. Hence, *Cochlospermum Planchoni *root augments spermatogenesis and attenuates cisplatin-induced oxidative stress through an antioxidant system of activities.

Furthermore, co-administration of vitamin C and cisplatin in this study as standard drug also ameliorates the toxic effects of cisplatin on testis but not as effective as *Cochlospermum planchonii *root extract as observed in our results since vitamin C is also a potent antioxidant (65), essential for humans and certain other animal species as a nutrient for a range of key metabolic reactions. Additionally, testicular environment contains vitamin C (66) and a number of other antioxidant molecules/systems for the maintenance of redox balance which is critical for optimal testicular function (67, 68) and this may explain the protective and beneficial effects of the vitamin in the testis. In this study, the inhibition of cisplatin-induced testicular toxicity by vitamin C may be due to its antioxidant property. We therefore suggest that the effects of cisplatin may be due to direct deleterious effects to the seminiferous tubules, probably via alteration of the oxidative status of the testicular milieu.

Moreover, cisplatin significantly interfere with development and maturation of male reproductive system, the rats in cisplatin treated group could not impregnate female rats when copulated with them. However the improvement of fertility in the groups co-administered with (cisplatin and vitamin C) and (cisplatin and *Cochlospermum planchonii* root extract) shows that vitamin C and *Cochlospermum planchonii* root extract as powerful antioxidant protect against the oxidative stress induced by cisplatin but *Cochlospermum planchonii *root extract is more effective. We therefore deduced from our findings that *Cochlospermum planchonii* improved sperm and hormone profile and also it has been reported that administration of *Cochlospermum planchonii* and vitamin C in rats improved sperm profile (45, 69) which is consistent with our findings.

## Conclusion

Cisplatin induced reproductive dysfunction at standard therapeutic dose levels can be protected by *Cochlospermum planchonii*. Concurrent administration of cisplatin and *Cochlospermum planchonii *could be encouraged to reduce the adverse reproductive effects of cisplatin when it is required for treatment of cancer in male.
